# Metal complexation-mediated stable and biocompatible nanoformulation of clinically approved near-infrared absorber for improved tumor targeting and photonic theranostics

**DOI:** 10.1186/s40580-021-00286-3

**Published:** 2021-11-10

**Authors:** Yong-Deok Lee, Hyeon Jeong Shin, Jounghyun Yoo, Gayoung Kim, Min-Kyoung Kang, Jae Jun Lee, Joona Bang, Jin-Kyoung Yang, Sehoon Kim

**Affiliations:** 1 Center for Theragnosis, Korea Institute of Science and Technology, Seoul, 02792 Republic of Korea; 2 Department of Chemical and Biological Engineering, Korea University, Seoul, 02841 Republic of Korea; 3 Laboratory Animal Center, KBIO Osong Medical Innovation Foundation, Osong, 28160 Republic of Korea; 4 KU-KIST Graduate School of Converging Science and Technology, Korea University, Seoul, 02841 Republic of Korea

**Keywords:** Indocyanine green, Metal complex, Dual-modal imaging, Photothermal therapy, Theranostics

## Abstract

Indocyanine green (ICG) is a clinically approved dye that has shown great promise as a phototheranostic material with fluorescent, photoacoustic and photothermal responses in the near-infrared region. However, it has certain limitations, such as poor photostability and non-specific binding to serum proteins, subjected to rapid clearance and decreased theranostic efficacy in vivo. This study reports stable and biocompatible nanoparticles of ICG (ICG-Fe NPs) where ICG is electrostatically complexed with an endogenously abundant metal ion (Fe^3+^) and subsequently nanoformulated with a clinically approved polymer surfactant, Pluronic F127. Under near-infrared laser irradiation, ICG-Fe NPs were found to be more effective for photothermal temperature elevation than free ICG molecules owing to the improved photostability. In addition, ICG-Fe NPs showed the markedly enhanced tumor targeting and visualization with photoacoustic/fluorescent signaling upon intravenous injection, attributed to the stable metal complexation that prevents ICG-Fe NPs from releasing free ICG before tumor targeting. Under dual-modal imaging guidance, ICG-Fe NPs could successfully potentiate photothermal therapy of cancer by applying near-infrared laser irradiation, holding potential as a promising nanomedicine composed of all biocompatible ingredients for clinically relevant phototheranostics.

**Supplementary Information:**

The online version contains supplementary material available at 10.1186/s40580-021-00286-3.

## Introduction

Photothermal therapy (PTT), a noninvasive light-triggered approach for cancer treatment, has attracted much attention owing to its effective therapeutic outcome with tumor specificity and minimal side effects compared to conventional chemotherapy and radiotherapy [1]. PTT exerts a localized therapeutic effect by generating heat through the nonradiative thermal relaxation of photoexcited light absorbers, denoted as photothermal agents (PTAs). In addition to its therapeutic effect, PTAs can serve as a contrast agent for photoacoustic (PA) imaging, as PA signals are mainly determined by photothermal conversion. Therefore, PTAs potentiate theranostics that combines multiple modalities of diagnostic imaging and therapy in a single platform to improve therapeutic outcomes through simultaneous disease treatment and monitoring. In recent years, a large variety of nanomaterial-based PTAs, such as gold nanostructures, carbon nanotubes, graphene, and transition metal sulfide nanoparticles, have been widely explored for PTT ablation [2–5]. Although these materials have shown high therapeutic efficacy in preclinical animal models, there are obvious concerns for their future clinical application because of their non-biodegradable nature and potential in vivo toxicity issues [6]. Therefore, a safe and highly effective PTA platform remains to be developed.

Indocyanine green (ICG), a US Food and Drug Administration (FDA)-approved contrast agent, has been extensively studied for the development of phototheranostic agents. ICG exhibits strong light absorption and fluorescence emission in the biologically transparent near-infrared (NIR) region (λ_abs, max_ = 780 nm, λ_em, max_ = 800 nm) [1, 7, 8]. In addition, ICG shows excellent light-to-heat conversion efficiency as well as a strong PA response [9–11]. These outstanding properties make ICG attractive as a PTA for biocompatible theranostic applications wherein NIR fluorescence and/or PA imaging have been combined with phototherapy by PTT. However, the theranostic use of ICG has been limited due to its poor chemical stability in biological environments, nonspecific tissue distribution, propensity to aggregate, poor photostability, and adsorption to serum proteins, resulting in rapid elimination from the body [12]. To overcome these limitations, various types of nanoparticles have recently been explored for encapsulating ICG, which can lead to enhanced stability and therapeutic efficacy [13–17]. Although the nanocarrier approach may offer a solution for future research and clinical use, limitations are still inevitable. The major issue is the easy leakage of free ICG from nanoparticles due to its amphiphilic nature, which can lead to a decrease in theranostic efficacy [18–20]. Chemical modification of ICG has also been studied to avoid unexpected leakage by increasing the hydrophobicity; however, chemical derivatization alters the intrinsic nature and identity of ICG and thus changes its FDA-approval status [19]. Therefore, it is crucial to develop an effective and clinically relevant strategy to prevent nanocarriers from releasing free ICG before disease targeting.

In this study, we developed an approach based on the metal complexation of ICG that allows for stable and biocompatible nanoformulation with enhanced disease targetability and theranostic performances. Iron ions (Fe^3+^), which are endogenously abundant in the biological environment, were employed as a cationic complexer for anionic ICG to prepare ICG-Fe complexes via the electrostatic interaction. The obtained ICG-Fe complex turned organic soluble with significantly reduced water solubility and thus able to be formulated with Pluronic F-127, an FDA-approved polymeric surfactant, to construct biocompatible phototheranostic ICG-Fe nanoparticles (ICG-Fe NPs) that are composed of all clinically usable or endogenous ingredients without chemical modification. The nanoformulated ICG-Fe complex was found to be chemically stable with enhanced photostability over free ICG in the physiological environment, to enhance the photothermal heating performance. NIR fluorescence (NIRF) imaging confirmed the improved in vivo tumor targetability compared to free ICG. The combined PA and NIRF imaging provided complementary cross-sectional and planar information on the ICG-Fe accumulated tumor tissue. Finally, we successfully applied ICG-Fe NPs for NIR laser-activated PTT of cancer, where only a single operation of PTT caused significance tumor volume suppression that was monitored by MRI imaging.

## Experimental

### Materials

All chemical reagents were purchased from commercial suppliers and were used without further purification. Pluronic F127, FeCl_3_, and dimethylsulfoxide (DMSO) were obtained from Sigma-Aldrich co. (St. Louis, Mo, USA). Indocyanine green (ICG) was purchased from TCI (Tokyo, Japan). RPMI 1640, DPBS, and fetal bovine serum (FBS) were purchased from WELGENE Inc. (Gyeongsan, Korea).

### Preparation of ICG-Fe NPs

ICG (30 mg) was dissolved in 2 mL of DMSO, and added to 20 mL aqueous solution of FeCl_3_ (20 mg). The resulting mixture was fully covered with aluminum foil to block light exposure and stirred at room temperature. After an hour, the precipitated ICG-Fe complex was washed several times with DW, re-dissolved in methanol, and then dried under vacuum. The obtained ICG-Fe complex was kept under vacuum before use. Next, ICG-Fe nanoparticles (ICG-Fe NPs) were fabricated using nanoprecipitation method. ICG-Fe complex (0.5 mg) was dissolved in 0.02 mL of DMSO, and ICG-Fe NPs were obtained by mixing it with 1 mL of pluronic F127 aqueous solution (10 mg/mL) using bath-sonicator for 2 min.

### Characterization

The size and morphology of ICG-Fe NPs were analyzed by transmission electron microscopy (TEM, Tecnai G2 F20, FEI, USA) after negative staining with 4% uranyl acetate. The optical properties were measured using a UV/Vis spectrometer (Agilent 8453 UV-Visible spectroscopy system, Agilent Technologies, USA) and a fluorescence spectrophotometer (Hitachi F-7000, Japan). FT-IR spectra were obtained by FT-IR spectrometer (Nicolet iS10, Thermo Fisher Scientific, USA). The amount of Fe^3+^ ions in ICG-Fe complex was determined with inductively coupled plasma optical emission spectroscopy (ICP-OES, iCAP6000, Thermo Fisher Scientific, USA). All in vivo/ex vivo images were acquired using IVIS spectrum imaging system (Caliper, PerkinElmer, USA). The photoacoustic images were taken by multispectral optoacoustic tomography (MSOT, iTheraMedical, Germany). MSOT measurements of various concentrations of ICG-Fe NPs and free ICG, from 0.023 to 0.375 mg/mL based on the ICG content, were acquired with excitation at 680–900 nm with 5 nm step size. Data was reconstructed using the software with back-projection algorithm.

### Measurement of temperature elevation

To verify the photothermal activity, 0.2 mL of DW, free ICG (0.375 mg/mL), and ICG-Fe NPs (0.5 mg/mL) in 96 well plate were irradiated with a NIR laser (785 nm, 1 W, Changchun New Industries Optoelectronics Tech. Co., Ltd., China) for 10 min, and the temperature change was measured by a thermocouple.

### Cell viability assay and in vitro PTT

Human colorectal adenocarcinoma cell line (HT-29) was purchased from ATCC, and cultured in RPMI 1640 supplemented with 10 % (v/v) FBS and 1% (v/v) antibiotics. HT-29 cells were seeded in 96 well plates and incubated overnight at 37 °C in humidified 5 % CO_2_ atmosphere. After being rinsed with PBS (pH 7.4), the cells were incubated with free ICG or ICG-Fe NPs at various concentrations for 24 h at 37 °C under the same conditions. For the PTT evaluation, cells were rinsed again with PBS, immersed in 100 µL of fresh culture medium, and subsequently illuminated using a 785 nm laser with energy density of 1 W for 1 min. To determine the relative cell viabilities, cells were washed with PBS, and a standard 3-(4,5-dimethyl-2-thiazolyl)-2,5-diphenyl-2-*H*-tetrazolium bromide (MTT) assay was used.

### Tumor-bearing mouse model

Animals are maintained under pathogen free condition in facility at Korea Institute of Science and Technology (KIST). The procedures were approved by the guidelines of the Institutional Animal care and use committee of KIST. Five week old male BALB/c nude mice were used to prepare tumor xenograft models. BALB/c tumor xenograft models are established by subcutaneous injection of 1 × 10^7^ HT-29 cells into the hind legs. HT-29 tumor bearing mice were used for imaging and PTT experiments about 2 weeks post-inoculation when the volume of tumors reached around 100 mm^3^.

###  In vivo toxicity

To confirm the in vivo toxicity of ICG-Fe NPs, normal mice were randomly divided in three groups (n = 3); i.e., PBS, free ICG and ICG-Fe NPs, which administrated for one time of intravenous injection. At 48 h after administration, blood sample and organs (liver and kidney) were collected for toxicity assessment.

###  In vivo imaging

Tumor-bearing mouse models were injected with 200 µL of PBS, free ICG, and ICG-Fe NPs (3.75 mg/kg ICG) via intravenous injection. After injection, time-dependent images were taken using the IVIS imaging system at 720/740 nm (ex/em) for fluorescence imaging, as well as the MSOT system for PA imaging. To ensure in vivo biodistribution of ICG-Fe NPs, another fluorescence dye (Cy5.5)-labeled ICG_Fe NPs were administered and imaged at 640 nm of excitation and 680 nm of emission. The Cy5.5-labeled ICG-Fe NPs were synthesized by using Cy5.5-conjugated F127. After in vivo imaging, the tumor and major organs including liver, spleen, kidney, heart and lung were dissected from the mouse 24 h post-injection and subjected to the ex vivo fluorescence examination.

###  In vivo PTT

The photoirradiation in the tumor site (785 nm, 1.5 W for 10 min) was applied 1 day after intravenous injection of PBS or ICG-Fe NPs (3.75 mg/kg ICG). The temperatures of the tumor tissues were recorded with an infrared thermal camera after laser irradiation. All animals were subjected to abdominal anesthesia with a Zoletil/Rompun mixture during PTT. To evaluate tumor size change precisely, *T*_*2*_-weighted MRI scanning was conducted before and 1 and 4 days after injection using BioSpec 47/40 (Bruker, Ettlingen, Germany).

## Results and discussion

### Synthesis and characterization of ICG-Fe NPs

**Fig. 1:**
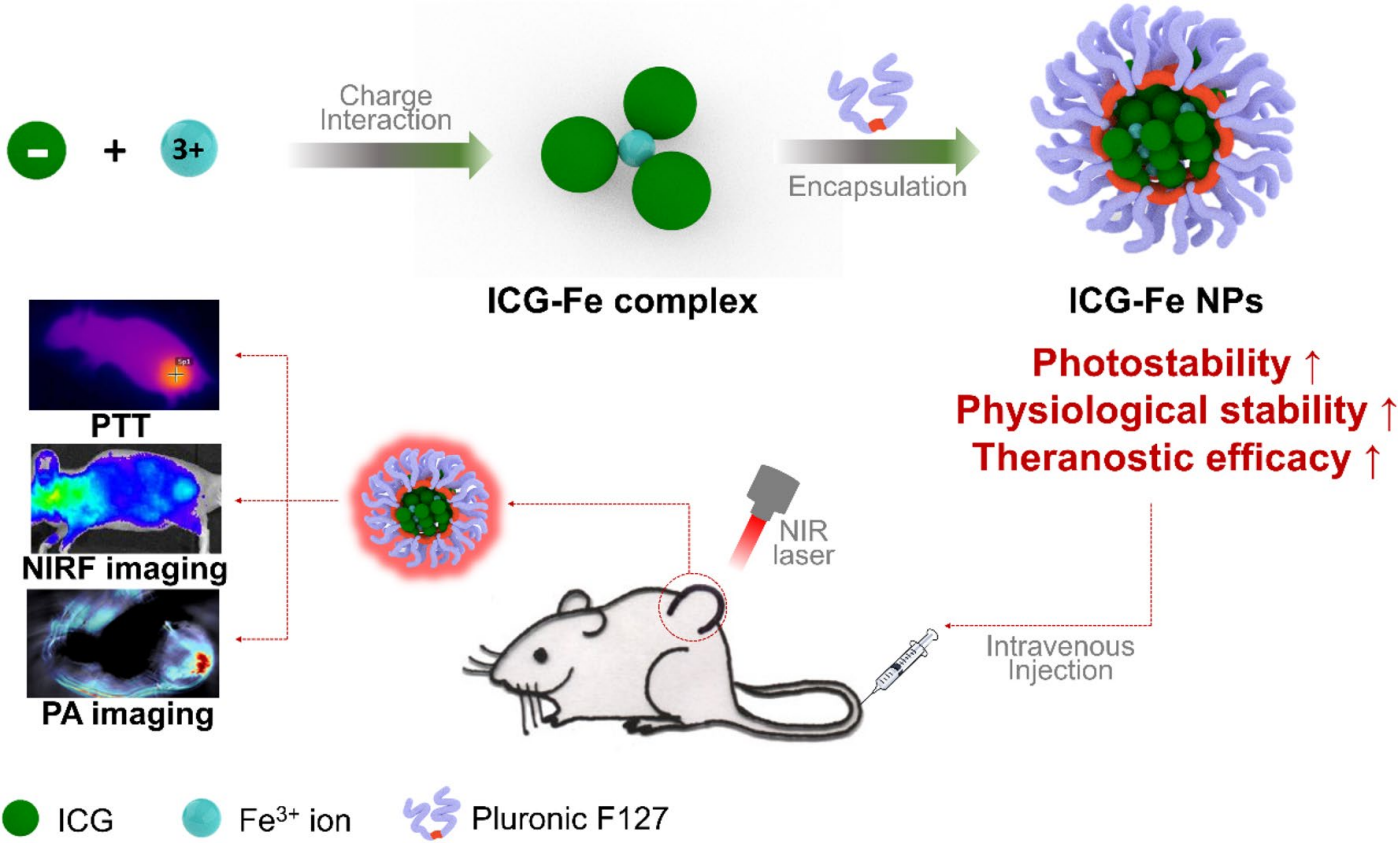
Schematic illustration of ICG-Fe NPs as theranostic nanoformulations for photonic theranostics

**Fig. 2:**
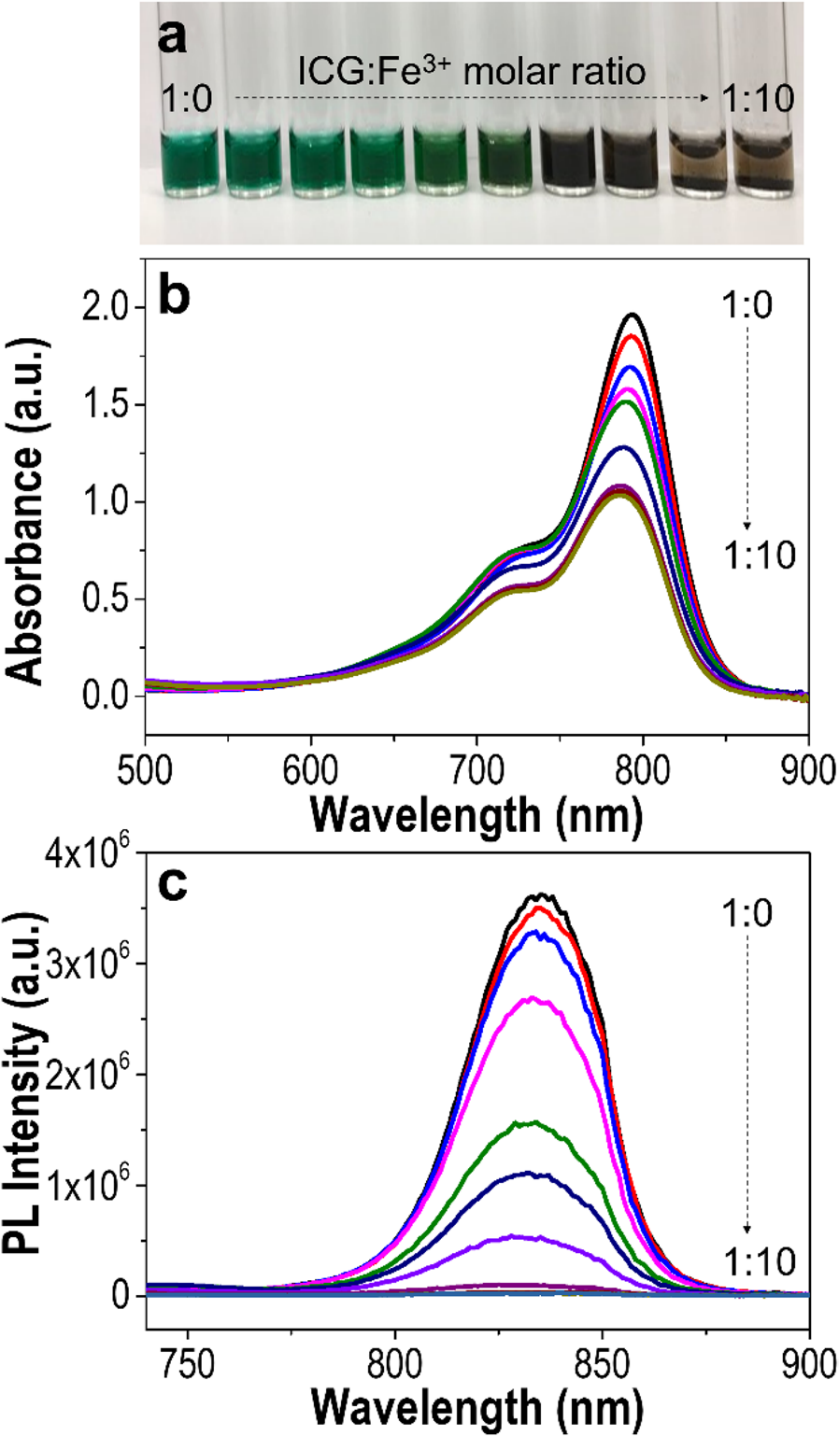
Characterization of the ICG-Fe complex. **a** Photograph of the ICG-Fe complex in water as a function of the ICG:Fe^3+^ molar ratio ranging from 1:0 to 1:10, and **b**, **c** their spectra in methanol: **b** UV/Vis absorption and **c** fluorescence emission (λ_ex_ = 720 nm)

The preparation process of ICG-Fe NPs is shown in Fig. 1. First, free ICG was reacted with Fe^3+^ ions in water to form a water-insoluble, hydrophobic ICG-Fe complex, implying that cationic Fe^3+^ ions and anionic ICG molecules were associated via the electrostatic interaction with their electric charges cancelled. As can be seen in Fig. 2a, the color of the ICG solution changed from green to brownish-black with sedimentation upon increasing the Fe^3+^ ion concentration, indicating the occurrence of complexation. The formation of the ICG-Fe complex was confirmed by absorption and fluorescence emission spectra (Fig. 2b, c). The absorption band at 793 nm and the fluorescence of ICG gradually decreased with increasing the molar ratio of Fe^3+^ from 1:0 to 1:10 (ICG:Fe^3+^). Upon increasing the Fe^3+^ ion concentration, the absorption band of free ICG shifted hypsochromically by ~8 nm, from 793 to 785 nm. The complex formed between the Fe^3+^ and ICG molecules was found to quench the fluorescence of ICG, likely owing to the paramagnetic property of Fe^3+^ [21–24]. This fluorescence quenching was further proved by the fluorescence lifetime as shown in Additional file 1: Figure S2. The fluorescence lifetime of ICG-Fe NPs was 0.94 ns, while free ICG exhibited 1.61 ns of fluorescence lifetime. The shortened fluorescence lifetime of ICG-Fe NPs supports that complexation between Fe^3+^ and ICG induced fluorescence quenching [25]. As the complex exhibited a higher quenching efficiency at the molar ratio of 1:10, we chose this condition for the metal-complexed ICG nanoformulation study. To characterize the composition of the ICG-Fe complex, FT-IR spectra of free ICG and the ICG-Fe complex were obtained (Additional file 1: Figure S1), where the ICG-Fe complex retained the characteristic peaks of free ICG near 1000, 1500, and 1400 cm^−1^, corresponding to the vinyl, C=C and S=O stretches, respectively [26]. Furthermore, the ICP-OES results showed that the parent counterion of free ICG (Na^+^) was almost replaced by Fe^3+^ with an ICG:Fe molar ratio of 3:1 (Additional file 1: Table S1).

**Fig. 3:**
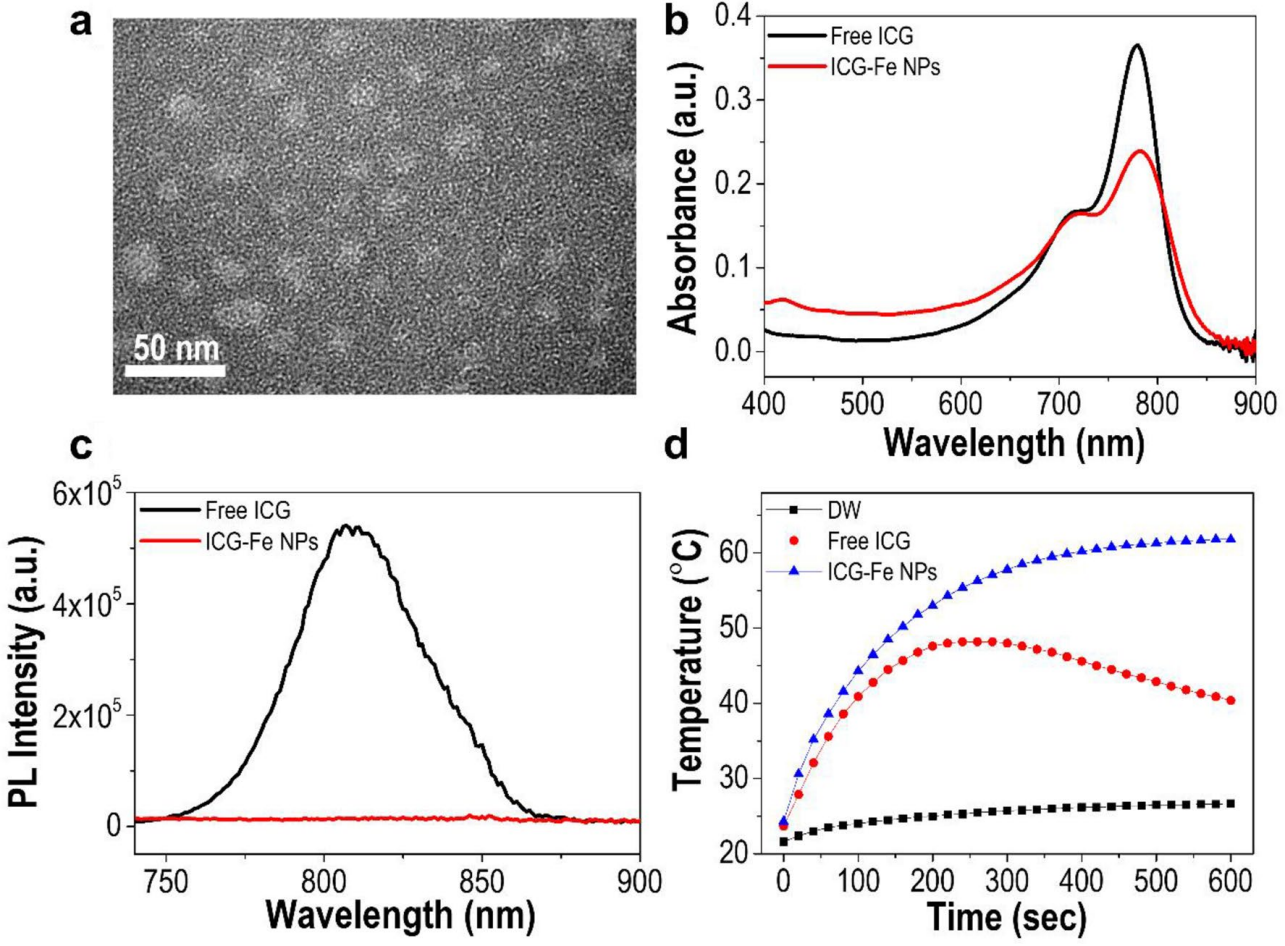
Characterization of ICG-Fe NPs. **a** TEM image. **b** UV/Vis absorption spectra and **c** fluorescence emission spectra of free ICG (black line) and ICG-Fe NPs (red line) in water. **d** Photothermal heating curve of pure water, free ICG, and ICG-Fe NPs over 10 min of laser irradiation (785 nm, 1 W). All experiments were conducted at the same concentration based on the ICG content

Next, ICG-Fe NPs were synthesized by encapsulating a hydrophobic ICG-Fe complex into a water-dispersed Pluronic F127 polymeric micelle. The transmission electron microscope (TEM) image indicated that the obtained ICG-Fe NPs were spherical in shape with an average diameter of 17.1 $\pm$ 2.6 nm (Fig. 3a). The nanoparticle loading of the ICG-Fe complex was also confirmed by UV/Vis absorption and fluorescence spectra (Fig. 3b, c). The absorption spectrum of ICG-Fe NPs was slightly red-shifted with broadening and an increased shoulder peak due to the formation of ICG dye aggregates in the nanoparticles [27–29]. Additionally, ICG-Fe NPs exhibited quenched fluorescence, which is similar to that of the ICG-Fe complex. These results indicate that the optical properties of the ICG-Fe complex were stably maintained after water-dispersed nanoformulation.

**Fig. 4:**
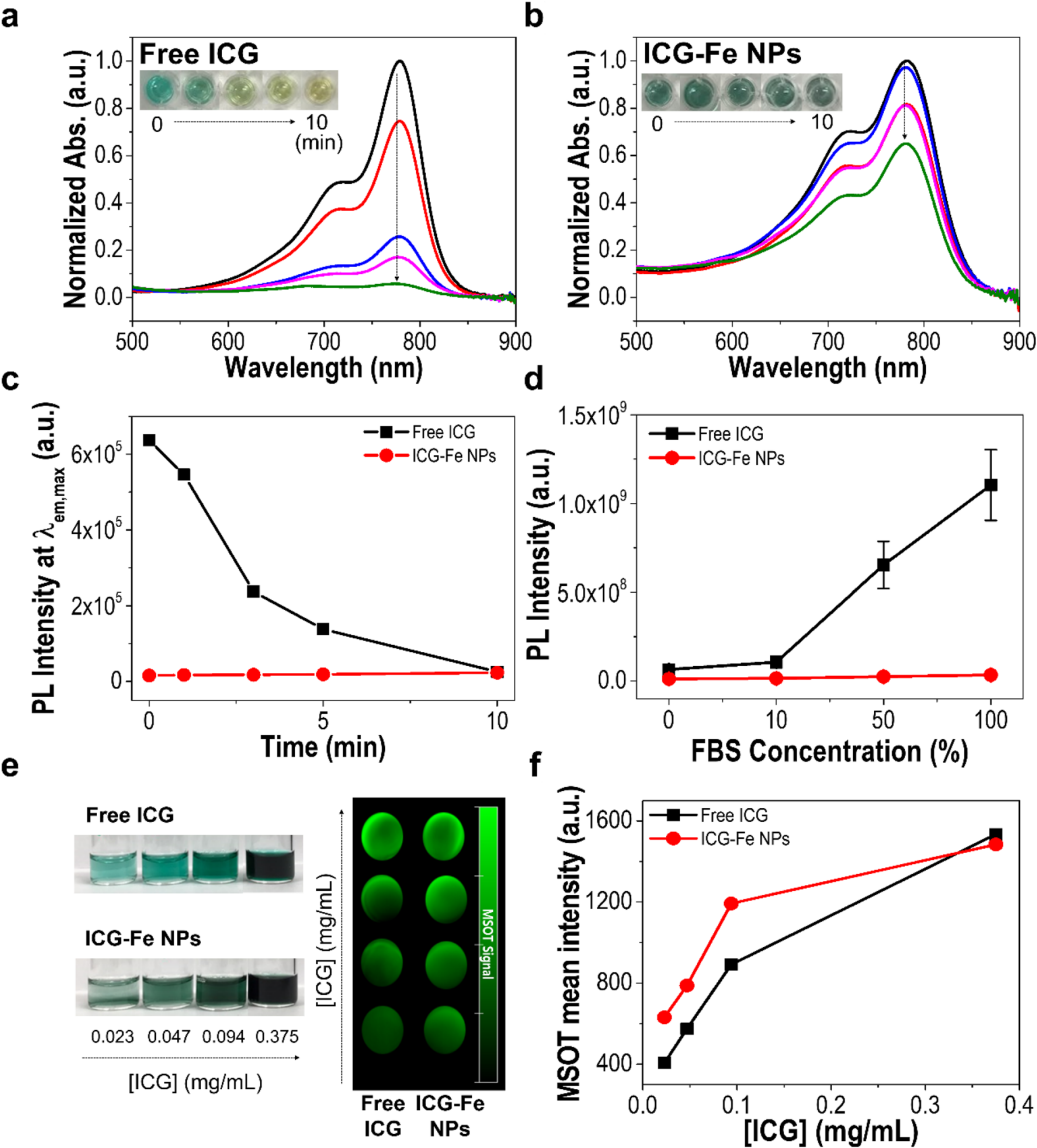
**a**-**c** Photostability of ICG-Fe NPs. UV/Vis absorption spectra of **a**) free ICG and (**b** ICG-Fe NPs (containing the same ICG content) depending on the laser irradiation time (785 nm, 1 W), normalized with the initial maximum absorbance; the insets present the representative photographs of free ICG and ICG-Fe NPs solution at each irradiation time. **c** PL intensity (at 820 nm) of free ICG and ICG-Fe NPs as a function of laser irradiation time (785 nm, 1 W). **d** Effect of FBS on the fluorescence intensity of free ICG and ICG-Fe NPs at 820 nm. **e** In vitro PA images and **f** mean PA intensities of free ICG and ICG-Fe NPs at different concentrations

The photothermal effect of ICG-Fe NPs was investigated using a 785 nm laser with an energy density of 1 W (Fig. 3d). The heating curves showed that ICG-Fe NPs readily reached over 60 °C within 10 min of laser irradiation. In contrast, free ICG increased the temperature up to 48.2 °C within 4 min, followed by a gradual decrease. ICG-Fe NPs showed a better temperature elevation efficiency than free ICG, despite reduced light absorption (Fig. 3b). To compare the photothermal heating performances between free ICG and ICG-Fe NPs, their optical properties were evaluated under laser irradiation. As shown in Fig. 4a–c, there was observed temporal declines in the UV/Vis absorbance and fluorescence emission in the case of free ICG, indicating significant photobleaching under the irradiation condition, whereas the photostability of ICG-Fe NPs was shown substantially enhanced. The enhanced photostability of ICG-Fe NPs is attributable to (1) the fluorescence quenching by paramagnetic Fe^3+^ to shorten the excited-state lifetime of ICG, and (2) nanoparticle encapsulation of ICG to disturb its contact to oxygen molecules, both of which might reduce the chance of photobleaching by photo-oxidation (reaction with oxygen molecules in the excited state). Along with photostability, we measured the stability of ICG-Fe NPs in the physiologically relevant FBS-containing medium. The emission intensities of free ICG and ICG-Fe NPs were evaluated depending on the concentration of FBS (Fig. 4d). The fluorescence intensity of free ICG significantly increased in proportion to FBS concentration as ICG was adsorbed to serum proteins to form more fluorescent ICG-protein complexes that are known to be promptly excreted from systemic circulation, being unfavorable for tumor-specific accumulation [12, 30, 31]. In sharp contrast, ICG-Fe NPs retained fluorescence quenching of the ICG-Fe complex regardless of FBS concentration, indicating the excellent protection of ICG from release and contact with the biological environment. In addition, the constant quenched state of ICG-Fe NPs in the biological environment can promote the non-radiative thermal decay, which can result in an increase in the PTT efficiency. Based on these results, we confirmed that ICG-Fe NPs confer considerable physiological/optical stability, being favorable for the improved tumor accumulation and PTT ability in vivo.

Next, we investigated the PA signaling properties of ICG-Fe NPs. As depicted in Fig. 4e, f, where PA signals were produced under laser irradiation and increased in proportion to their concentration from 0.023 to 0.375 mg/mL based on the ICG content. Although ICG-Fe NPs presented only a similar level of PA signals to those of free ICG under the given condition, it is anticipated that their enhanced photo and physiological stability over time would be beneficial for tumor-specific PA imaging in vivo.

###  In vitro phototoxic property

**Fig. 5:**
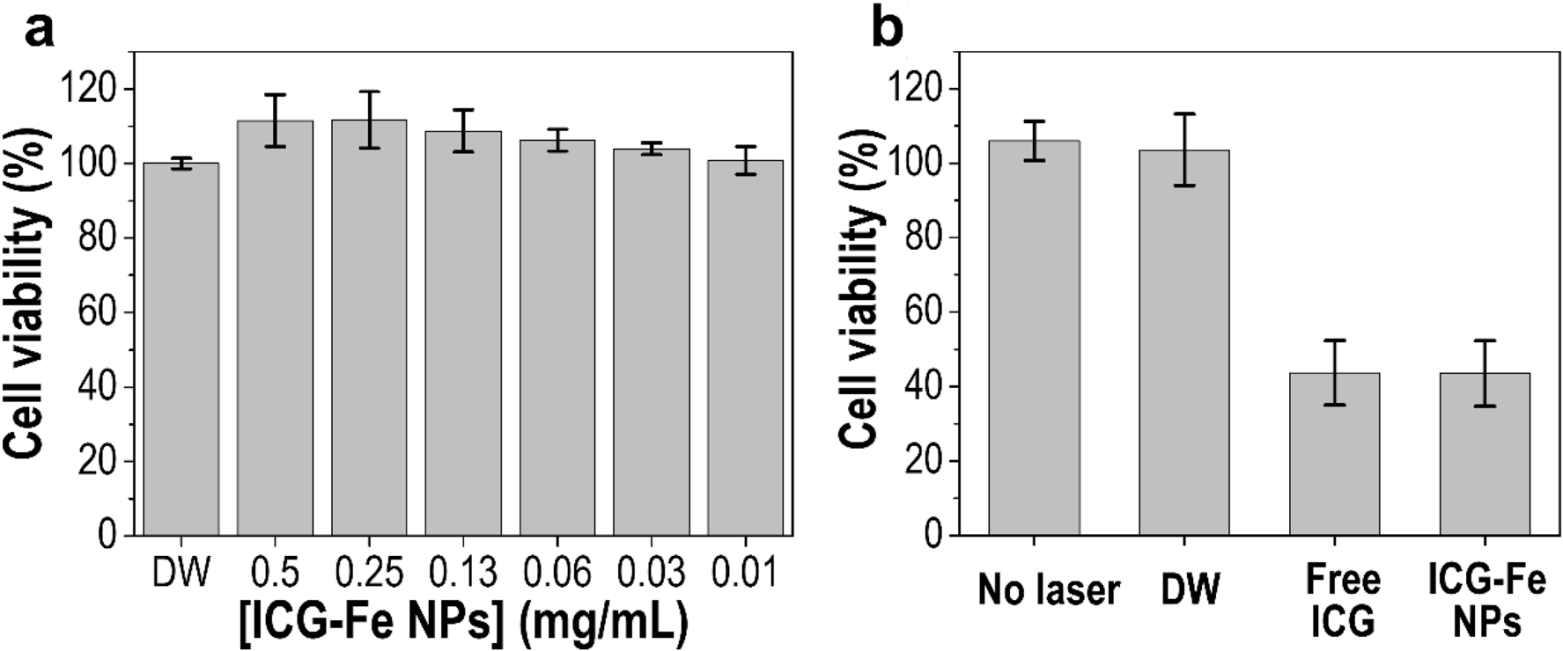
**a** In vitro dark toxicity of ICG-Fe NPs against HT-29 cells incubated at various concentrations for 24 h. **b** In vitro phototoxicity of DW, free ICG (0.375 mg/mL), and ICG-Fe NPs (0.5 mg/mL) under 785 nm laser irradiation (5 min, 1 W). The data are shown as the mean ± SD (*n* = 5)

The intrinsic cytotoxicity of ICG-Fe NPs was examined using an MTT assay in HT-29 cells. As shown in Fig. 5a, no obvious cytotoxicity was observed after the incubation of the cells with ICG-Fe NPs at different concentrations for 24 h, indicating that ICG-Fe NPs are biocompatible. In addition, the photothermally induced cytotoxicity by ICG-Fe NPs was evaluated after irradiation with the 785 nm laser at a power density of 1 W for 5 min (Fig. 5b). The laser-alone treatment exhibited no cytotoxicity; however, free ICG and ICG-Fe NPs under the given irradiation condition induced considerable cell death of over 50%. No significant difference was found between free ICG and ICG-Fe NPs, likely because in both cases, the temperature elevations were shown to be similar during the initial 5 min of laser irradiation (Fig. 3d). These results conclude that ICG-Fe NPs are safe in the absence of light but able to be cytotoxic under light due to the efficient photothermal heating property, being useful for PTT.

### PA and NIRF dual-modal tumor imaging in vivo

**Fig. 6:**
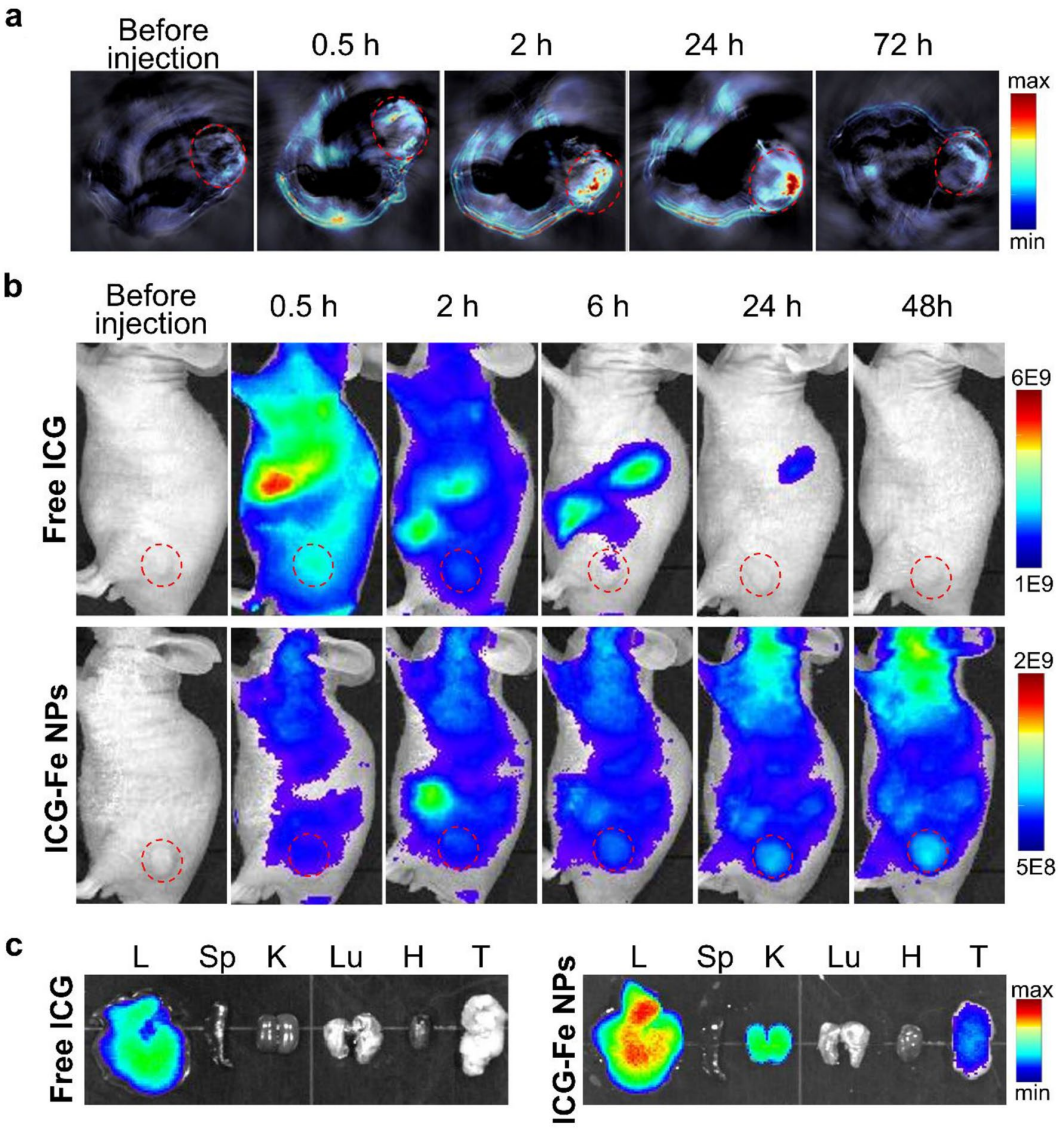
In vivo dual-modal imaging. **a** Cross-sectional PA images of the HT-29 tumor xenograft in a mouse model after intravenous injection of ICG-Fe NPs ([ICG] = 3.75 mg/kg). **b** In vivo and **c** ex vivo fluorescence images of a HT-29 tumor-bearing mouse after intravenous injection of free ICG and ICG-Fe NPs. Excitation (740/30 nm) and emission (790/20 nm) filters were used for fluorescence imaging. The red dotted circles in **b** indicate the tumor region. Tumors and other organs were collected 24 h post injection: L: liver, Sp: spleen, K: kidney, Lu: lung, H: heart, T: tumor

The feasibility of ICG-Fe NPs for in vivo PA and NIRF dual-modal imaging was evaluated in an HT-29 tumor-bearing mouse model. When the size of the tumor reached approximately 100 mm^3^, the mice were intravenously injected with ICG-Fe NPs, which were imaged using MSOT and IVIS systems. As shown in Fig. 6a, a low PA signal was detected before the injection of ICG-Fe NPs, whereas after the administration of ICG-Fe NPs, the tumor manifested an obvious PA signal that was the strongest 24 h post-injection. Meanwhile, the NIRF signal was also accumulated at the tumor with time after the injection of ICG-Fe NPs (Fig. 6b). In the case of free ICG-treated mice, however, the fluorescence signal was initially distributed throughout the body with the highest accumulation at the liver rather than the tumor, and as expected, cleared from the body much faster than ICG-Fe NPs. Since ICG-Fe NPs showed relatively low fluorescence signals compared with free ICG owing to the fluorescence quenching, we also conducted the in vivo tumor imaging with the injection of Cy5.5-labeled ICG-Fe NPs (Additional file 1: Figure S3), which confirmed similar tumor accumulation and in vivo distribution with cy5.5 fluorescence 2 h post-injection. For further validation, the organs and tumors were harvested 24 h post-injection. As presented in Fig. 6c, the majority of free ICG was present in the liver, whereas ICG-Fe NPs exhibited substantial accumulation in the tumor, liver, and kidney. These results clearly suggest that ICG-Fe NPs can significantly improve the tumor targetability via the enhanced permeability and retention (EPR) effect with reduced body clearance compared with free ICG, to potentiate dual-modal tumor imaging via the systemic targeting of ICG-Fe NPs.

###  In vivo PTT of cancer

**Fig. 7:**
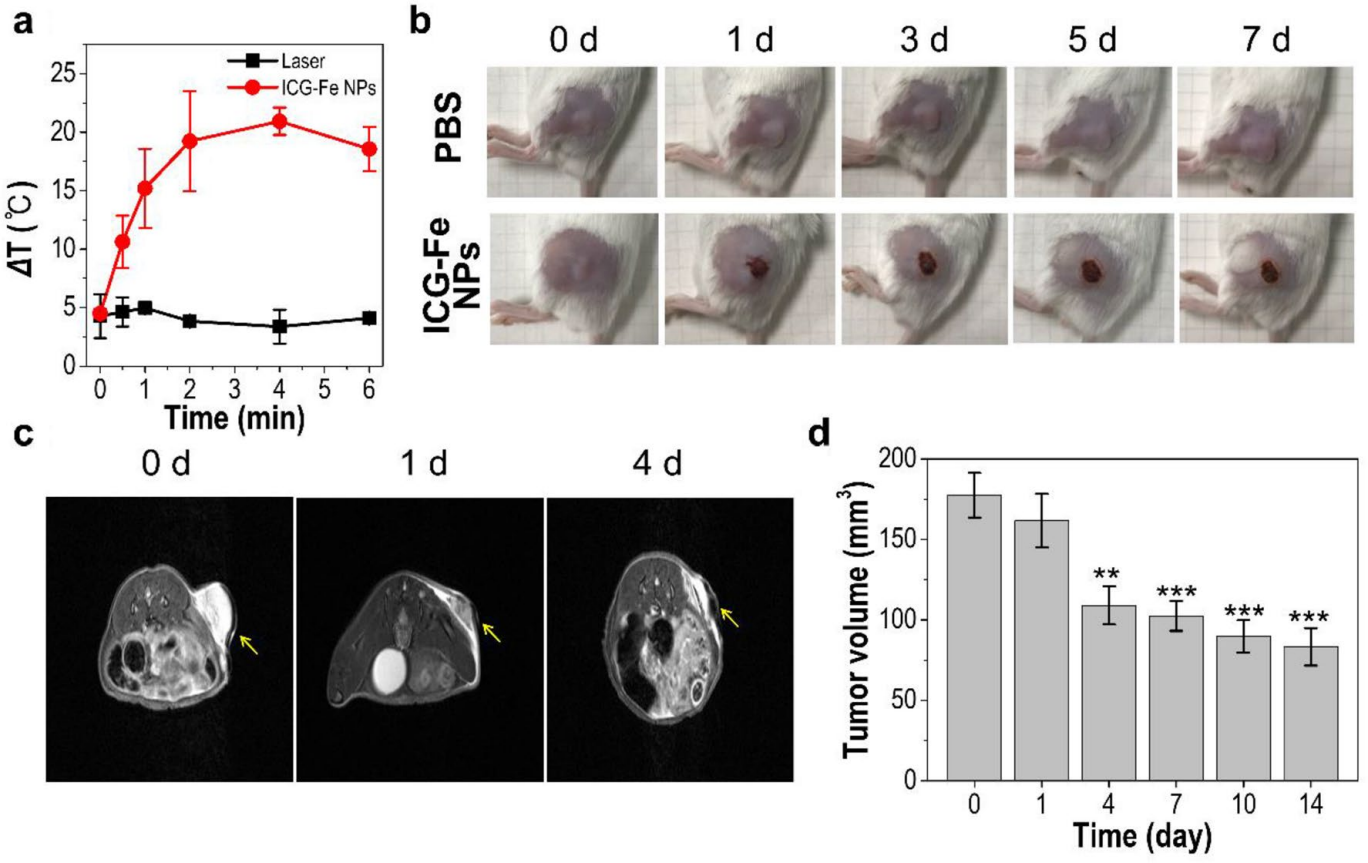
In vivo PTT in a HT-29 tumor-bearing mouse model. **a** Temperature changes at the tumor during treatment with laser alone or ICG-Fe NPs + laser. **b** Photographs of HT-29 tumor-bearing mice treated with PBS or ICG-Fe NPs + laser. **c** Representative axial in vivo T_*2*_-weighted MRI images of mice treated with ICG-Fe NPs + laser. **d** Tumor volume changes estimated via MRI for 14 days post-treatment. Laser irradiation (785 nm, 1.5 W) was performed for 10 min 24 h after intravenous injection. The yellow arrow indicates a tumor region. Data are means ± SD based on 3 mice per group. **P < 0.01; ***P < 0.001 (n = 3) using t-test

The PTT efficacy of ICG-Fe NPs was investigated in mouse models whose tumor region was exposed to irradiation for 10 min after 24 h of ICG-Fe NP injection. When monitored with an infrared thermal camera, the ICG-Fe NP-treated tumor gradually increased the temperature up to 56.4 °C within 4 min after laser irradiation, exceeding the destructive threshold for irreversible tumor ablation, while the laser-alone treatment did not show any temperature increase under the given irradiation condition (Fig. 7a). After PTT, a therapeutic response was observed. As shown in Fig. 7b, mice treated with PBS alone showed sustained tumor growth. In stark contrast, tumor growth was considerably suppressed with obvious signs of necrosis, such as redness and scabbing on the tumor surface in the ICG-Fe NP-treated group under laser irradiation, indicating the outcome of efficient PTT. For a more rigorous study on tumor size reduction, we conducted MRI analysis of the ICG-Fe NP-treated mice after PTT. MRI images and estimated tumor volume showed that the tumor appeared to be destructive over time; in particular, there was a statistically significant shrinkage in the tumor volume from 4 days to 14 days after treatment, suggesting that systemically targeted ICG-Fe NPs exert a remarkable therapeutic impact for PTT of cancer (Fig. 7c, d).

Histological examination of the major organs was conducted to evaluate the in vivo toxicity of ICG-Fe NPs. H&E staining images of liver and kidney collected from ICG-Fe NP-treated mice revealed no obvious tissue damage compared with those of the control group (Additional file 1: Figure S4). Furthermore, liver function parameters of the ICG-Fe NP-treated group, including aspartate aminotransferase and alanine aminotransferase, were measured within the normal range (Additional file 1: Table S2). In addition, the body weight did not obviously change during cancer PTT after intravenous injection of ICG-Fe NPs (Additional file 1: Figure S5). This demonstrates that ICG-Fe NPs hold potential as a biocompatible theranostic agent with minimal in vivo toxicity.

## Conclusions

In summary, ICG-Fe NPs were synthesized as a theranostic nanoagent for PA/NIRF dual-modal imaging and PTT treatment of cancer. The ICG-Fe complex and its nanoformulation promoted photostability under laser irradiation, which greatly enhanced PTT efficiency. Moreover, ICG-Fe NPs demonstrated efficient tumor accumulation in vivo with a reduced clearance rate and increased physiological stability. The PA/NIRF dual-modal imaging and cancer PTT performances, along with suitable biocompatibility in vitro and in vivo, demonstrated the clinically relevant theranostic potential of ICG-Fe NPs for dual-modal imaging-guided phototherapy of cancer.

## Supplementary Information

**Additional file 1: Figure S1**. FT-IR spectra of free ICG (black line) and ICG-Fe complex (red line). **Figure S2.** Fluorescence lifetime decay curves of free ICG and ICG-Fe NPs (100 μM) dissolved in DMSO. **Table S1**. Elemental analysis performed by ICP-OES of free ICGand ICG-Fe NPs. **Figure S3**. In vivo fluorescence images of a HT-29 tumor-bearing mouse after intravenous injection ofcy5.5-labeled ICG-Fe NPs. Excitation (640 nm) and emission (710 nm) filters were used for the fluorescence images. The red dotted circles indicate a tumor region. **Figure S4**. Representative histological images of lung and kidney stained with haematoxylin and eosin (H&E). Scalebar: 100 μm.**Table S2**. Effect of ICG-Fe NPs on liver function markers. **Figure S5**. Body weights of mice during14 day after intravenous injection of ICG-Fe NPs and PTT

## Data Availability

Not applicable.
