# Supplementary material for: Metal complexation-mediated stable and biocompatible nanoformulation of clinically approved near-infrared absorber for improved tumor targeting and photonic theranostics
Source: Nano Converg. 2021 Nov 10;8:36. doi: 10.1186/s40580-021-00286-3 (PMC8581101; doi:10.1186/s40580-021-00286-3)
Supplement: Supplementary file 1 — Additional file 1: Figure S1. FT-IR spectra of free ICG (black line) and ICG-Fe complex (red line). Figure S2. Fluorescence lifetime decay curves of free ICG and ICG-Fe NPs (100 μM) dissolved in DMSO. Table S1. Elemental analysis performed by ICP-OES of free ICGand ICG-Fe NPs. Figure S3. In vivo fluorescence images of a HT-29 tumor-bearing mouse after intravenous injection ofcy5.5-labeled ICG-Fe NPs. Excitation (640 nm) and emission (710 nm) filters were used for the fluorescence images. The red dotted circles indicate a tumor region. Figure S4. Representative histological images of lung and kidney stained with haematoxylin and eosin (H&E). Scalebar: 100 μm.Table S2. Effect of ICG-Fe NPs on liver function markers. Figure S5. Body weights of mice during14 day after intravenous injection of ICG-Fe NPs and PTT [file 40580_2021_286_MOESM1_ESM.docx]

**Additional file 1**

**Metal Complexation-Mediated Stable and Biocompatible Nanoformulation of Clinically Approved Near-Infrared Absorber for Improved Tumor Targeting and Photonic Theranostics**

Yong-Deok Lee^1,2,†^, Hyeon Jeong Shin^1,†^, Jounghyun Yoo^1,†^, Gayoung Kim^1^, Min-Kyoung Kang^3^, Jae Jun Lee^3^, Joona Bang^2,^*, Jin-Kyoung Yang^1,^*, Sehoon Kim^1,4,^*

**Preparation of Cy5.5-labeled ICG-Fe NPs**

Cy5.5-labeled ICG-Fe NPs were prepared by encapsulating ICG-Fe complex within Cy5.5-labeled Pluronic F127 (Cy5.5-F127). For synthesis of Cy5.5-F127, vinylsulfone-functionalized Cy5.5 (FPR-675, BioActs Co. Ltd., Korea) was conjugated to the hydroxyl group of F127 by mixing F127 (100 mg) and FPR-675 (0.5 mg) in PBS (pH 8.0) for 2 h at room temperature. As-prepared Cy5.5-F127 was dialyzed with Spectra/Por membrane® (MWCO: 3.5 kDa) in water 3 d and then lyophilized. Next, Cy5.5-labeled ICG-Fe NPs were fabricated as described in the manuscript.


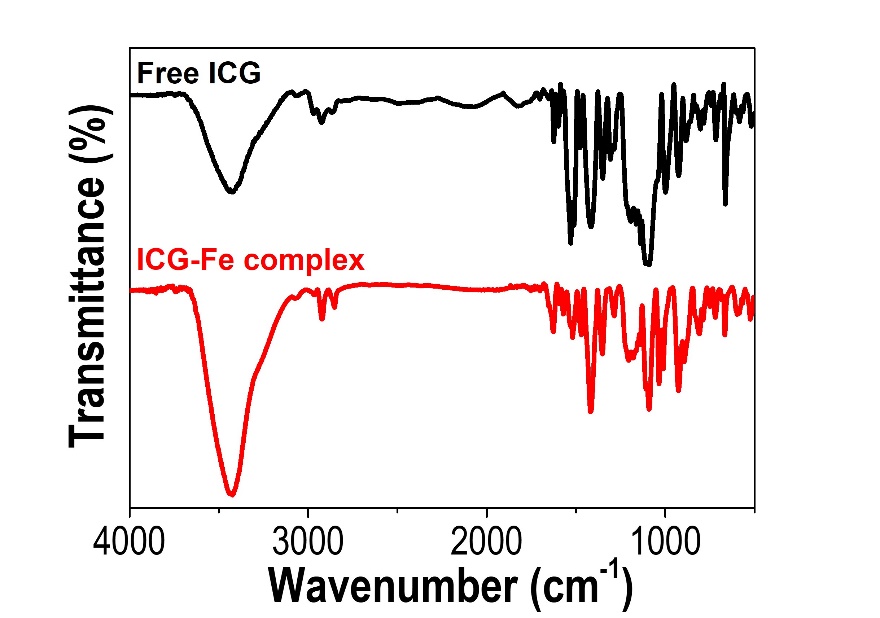


**Figure S1**. FT-IR spectra of free ICG (black line) and ICG-Fe complex (red line).

**Figure S2.** Fluorescence lifetime decay curves of free ICG and ICG-Fe NPs (100 μM) dissolved in DMSO.

**Table S1**. Elemental analysis performed by ICP-OES of free ICG and ICG-Fe NPs.

|  | **Na (wt%)** | **Fe (wt%)** |
| --- | --- | --- |
| Free ICG | 4.87 | < 0.2 |
| ICG-Fe NPs | < 0.1 | 1.89 |


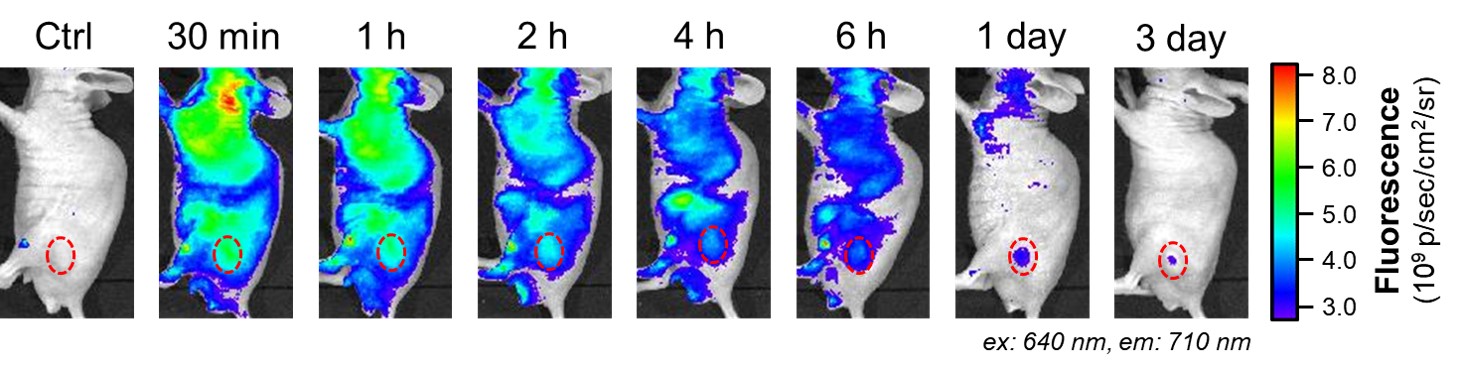


**Figure S3**. *In vivo* ﬂuorescence images of a HT-29 tumor-bearing mouse after intravenous injection of cy5.5-labeled ICG-Fe NPs. Excitation (640 nm) and emission (710 nm) ﬁlters were used for the ﬂuorescence images. The red dotted circles indicate a tumor region.


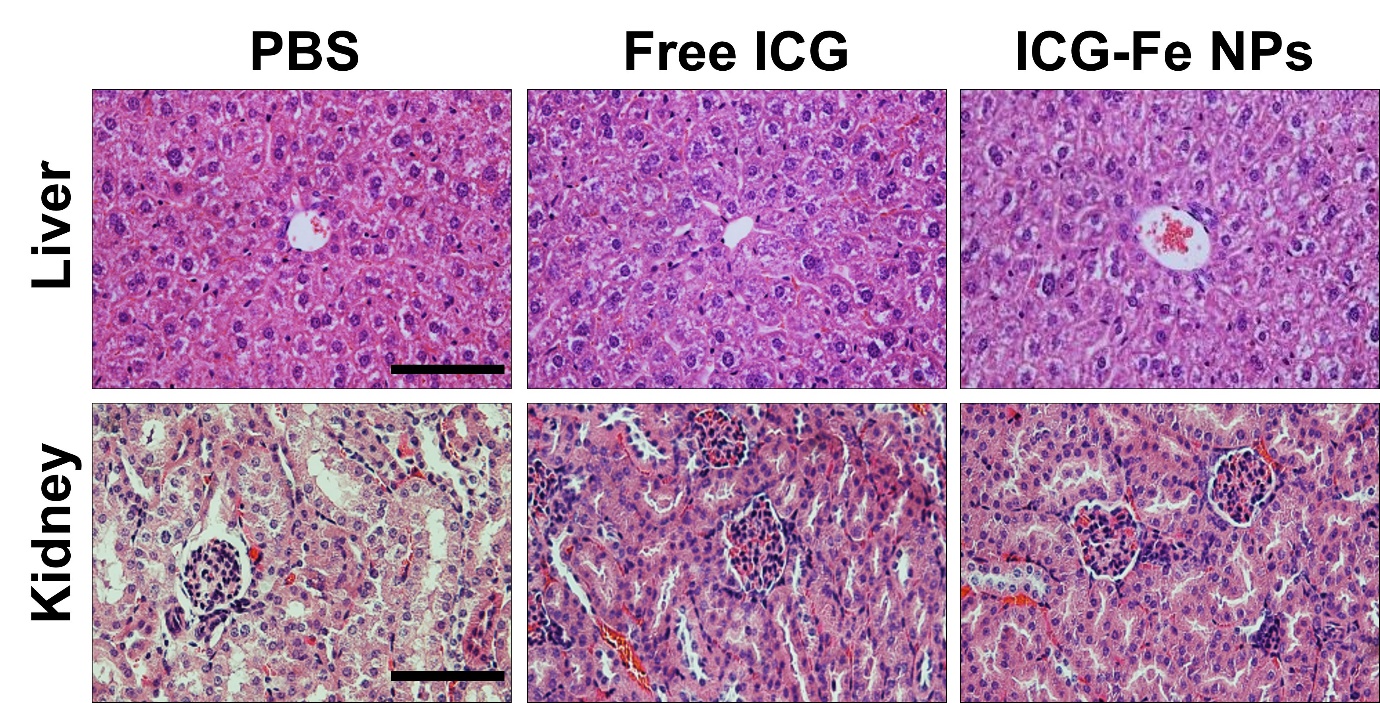


**Figure S4**. Representative histological images of lung and kidney stained with haematoxylin and eosin (H&E). Scale bar: 100 μm.

**Table S2**. Effect of ICG-Fe NPs on liver function markers.

| **Group** | **AST level (U/L)^a^** | **ALT level (U/L)^b^** |
| --- | --- | --- |
| PBS | 114.9 ± 0.9 | 54.8 ± 1.4 |
| Free ICG | 143.4 ± 2.0 | 56.5 ± 1.2 |
| ICG-Fe NPs | 87.3 ± 1.1 | 48.4 ± 1.5 |

AST: Aspartate aminotransferase; ALT: Alanine aminotransferase

^a^Normal range: 54-298 U/L

^b^Normal range: 17-77 U/L

**Figure S5.** Body weights of mice during 14 day after intravenous injection of ICG-Fe NPs and PTT.
